# Effectiveness of face masks for reducing transmission of SARS-CoV-2: a rapid systematic review

**DOI:** 10.1098/rsta.2023.0133

**Published:** 2023-10-09

**Authors:** Leah Boulos, Janet A. Curran, Allyson Gallant, Helen Wong, Catherine Johnson, Alannah Delahunty-Pike, Lynora Saxinger, Derek Chu, Jeannette Comeau, Trudy Flynn, Julie Clegg, Christopher Dye

**Affiliations:** ^1^ Maritime SPOR SUPPORT Unit, Nova Scotia Health, 5790 University Avenue, Halifax, Nova Scotia B3H 1V7, Canada; ^2^ IWK Health Centre, 5980 University Avenue, Halifax, Nova Scotia B3K 6R8, Canada; ^3^ School of Nursing, Dalhousie University, 6299 South Street, Halifax, Nova Scotia B3H 4R2, Canada; ^4^ Faculty of Health, Dalhousie University, 6299 South Street, Halifax, Nova Scotia B3H 4R2, Canada; ^5^ Division of Infectious Diseases, Dalhousie University, 6299 South Street, Halifax, Nova Scotia B3H 4R2, Canada; ^6^ Department of Health and Rehabilitation Services, Western University, 1151 Richmond Street, London, Ontario N6A 3K7, Canada; ^7^ Division of Infectious Diseases, Departments of Medicine and Medical Microbiology and Immunology, University of Alberta, 116 Street & 85 Avenue, Edmonton, Alberta T6G 2R3, Canada; ^8^ Department of Health Research Methods, Evidence and Impact, McMaster University, 1280 Main Street West, Hamilton, Ontario L8S 4L8, Canada; ^9^ Department of Medicine, McMaster University, 1280 Main Street West, Hamilton, Ontario L8S 4L8, Canada; ^10^ The Research Institute of St Joe's Hamilton, St Joseph's Healthcare Hamilton, 50 Charlton Avenue East, Hamilton, Ontario L8N A46, Canada; ^11^ Patient/Public Partner, University of Oxford, 11A Mansfield Road, Oxford OX1 3SZ, UK; ^12^ Department of Biology, University of Oxford, 11A Mansfield Road, Oxford OX1 3SZ, UK

**Keywords:** SARS-CoV-2, COVID-19, face masks, respirators, systematic review, rapid review

## Abstract

This rapid systematic review of evidence asks whether (i) wearing a face mask, (ii) one type of mask over another and (iii) mandatory mask policies can reduce the transmission of SARS-CoV-2 infection, either in community-based or healthcare settings. A search of studies published 1 January 2020–27 January 2023 yielded 5185 unique records. Due to a paucity of randomized controlled trials (RCTs), observational studies were included in the analysis. We analysed 35 studies in community settings (three RCTs and 32 observational) and 40 in healthcare settings (one RCT and 39 observational). Ninety-five per cent of studies included were conducted before highly transmissible Omicron variants emerged. Ninety-one per cent of observational studies were at ‘critical’ risk of bias (ROB) in at least one domain, often failing to separate the effects of masks from concurrent interventions. More studies found that masks (*n* = 39/47; 83%) and mask mandates (*n* = 16/18; 89%) reduced infection than found no effect (*n* = 8/65; 12%) or favoured controls (*n* = 1/65; 2%). Seven observational studies found that respirators were more protective than surgical masks, while five found no statistically significant difference between the two mask types. Despite the ROB, and allowing for uncertain and variable efficacy, we conclude that wearing masks, wearing higher quality masks (respirators), and mask mandates generally reduced SARS-CoV-2 transmission in these study populations.

This article is part of the theme issue 'The effectiveness of non-pharmaceutical interventions on the COVID-19 pandemic: the evidence'.

## 1. Introduction

Recommendations and mandatory policies to use medical or surgical masks, respirators such as N95, KN95 or FFP2 masks, and other facial coverings such as cloth masks have been commonly implemented among non-pharmaceutical interventions (NPIs) during the COVID-19 pandemic. Initially implemented in healthcare settings, mask recommendations and mandates for members of the public became more common globally as the pandemic progressed through 2020 and 2021. Although mask mandates had been discontinued in most jurisdictions by 2023, there remains a need to critically examine the role of masks among other NPIs in reducing the transmission of SARS-CoV-2 and other respiratory pathogens in preparation for future surges of COVID-19 or new epidemics.

Previous systematic reviews have examined evidence of the effectiveness of mask-wearing against respiratory viruses with varied conclusions, reflecting a lack of high-quality or conclusive data and heterogeneous methods of investigation. A living rapid review by Chou *et al*. [1] included randomized controlled trials (RCTs) and observational studies with a direct focus on COVID-19. At its last (eighth) update, the authors found low-to-moderate strength evidence supporting the benefit of mask use to prevent SARS-CoV-2 infection in the community. However, the review also concluded that there was insufficient evidence to recommend masks in healthcare settings. In an early-pandemic systematic review of COVID-19 and non-COVID-19-focused observational studies, Chu *et al*. [2] found evidence of low certainty to suggest that ‘face mask use could result in a large reduction in risk of infection’.

Recently, Jefferson *et al*. [3] completed a fifth revision of their 2006 Cochrane review of physical interventions (screening, isolation, quarantine physical distancing, face masks and handwashing) to interrupt or reduce the spread of respiratory viruses. The review included 78 RCTs in community or healthcare settings. Many of the trials were conducted during non-epidemic influenza periods with only six new trials (two focused on masks) conducted during the COVID-19 pandemic. The authors reported low to moderate certainty evidence on the effects of masks on the spread of influenza-like illness or COVID-19-like illness. They concluded that the high risk of bias (ROB) in the trials, variations in outcome measures and adherence to interventions during the studies hindered their ability to draw firm conclusions. In an earlier, pre-COVID-19 edition of their review, which included case–control and other observational studies, Jefferson *et al*. [4] concluded that implementing transmission barriers, isolation and hygienic measures are effective at containing respiratory virus epidemics, and that surgical masks or N95 respirators were the most consistent and comprehensive supportive measures.

These are the most important and prominent reviews, among a large number of reviews of variable quality, which inevitably include many of the same studies. The review that was limited to RCTs was unable to draw firm conclusions about the effectiveness of masks, whereas those that included observational studies were able to draw low to moderate strength conclusions that generally favoured the effectiveness of masks.

Due to the difficulty in performing rigorous RCTs to assess NPIs and the relative urgency to collect data of all kinds during the pandemic, observational studies have been the most common type of research to address the effectiveness of masks. Therefore, as in Chou *et al*. and Chu *et al*.'s work, this review includes a synthesis of observational data in addition to RCT data on the effectiveness of masks, different types of masks and mask mandates for reducing transmission of SARS-CoV-2. We have taken an inclusive approach, acknowledging the reduced certainty of conclusions drawn from observational studies, particularly studies done during a time of polarized opinion on NPIs, and when pressures on peer review led to greater variability in the quality of published studies [5,6].

### (a) Research question

Our primary question was: What is the best available evidence about the effectiveness of masks in reducing transmission of SARS-CoV-2 in community-based and healthcare settings?

We also asked two subsidiary questions:

1. What is the best available evidence about which types of masks (respirators, surgical masks or other face coverings such as cloth masks) are the most effective at reducing transmission of SARS-CoV-2 in community-based and healthcare settings?
2. What is the best available evidence about the effectiveness of mandatory masking policies in reducing transmission of SARS-CoV-2 in community-based and healthcare settings?

## 2. Methods

This rapid review follows guidelines set out by the Joanna Briggs Institute for systematic reviews [7], with adjustments made due to its rapid timeline guided by Straus *et al*.'s guide to rapid reviews for policymakers [8].

In this review a ‘surgical mask’ is a multi-layer polypropylene mask as used in medical and surgical healthcare settings, a ‘cloth mask’ is a face covering of variable manufacture that covers the mouth and nose, and a ‘respirator’ is a polypropylene mask manufactured for higher filtration efficiency which is usually intended to be fitted to the wearer.

### (a) Search strategy

The search was designed by the review team in consultation with a health sciences librarian, who finalized, translated and executed the search in PubMed (NCBI), iCite (NCBI), Embase (Ovid), CINAHL (EBSCOhost) and ERIC (ProQuest). The search terms limited results to studies published from 1 January 2020 in English. Keywords and controlled vocabulary terms related to SARS-CoV-2, COVID-19 and masks were combined with search filters to limit results to reviews, RCTs, quasi-experimental studies or cohort studies of SARS-CoV-2 transmission, and to exclude animal-only studies. Relevant preprints indexed in PubMed, iCite and Embase were retrieved by the search; no other preprint servers or trial registers were searched. Searches were initially executed on 25 November 2022 and updated weekly up to and including 27 January 2023. Search results were exported to EndNote for deduplication, then imported to Covidence (Veritas Health Innovation, Melbourne, Australia; www.covidence.org) for screening by the review team. The full search strategy in PubMed is included in the electronic supplementary material, Appendix S1.

### (b) Source selection criteria

We included studies if they were written in English, reported on COVID-19 alone or in combination with other respiratory infectious diseases, and reported on masks as a method of reducing or preventing transmission of COVID-19. Included studies could be based either in the community or in a healthcare setting. RCTs, non-RCTs, quasi-experimental studies and observational studies (e.g. case–control studies, prospective or retrospective cohort studies) with a comparison group were included, whether published or preprint.

We excluded studies if they did not involve human participants, if they did not report mask-related data separately from other interventions, or if they relied on self-reported SARS-CoV-2 status of participants. We excluded studies that compared mask-wearing and COVID-19 infection in large groups (sometimes called ‘ecological studies’), for example across whole countries or across multiple countries, because of the high risk of confounding factors. Modelling studies, mechanical studies, laboratory-based studies, descriptive studies, prevalence studies, conference abstracts and non-studies (e.g. press releases, commentaries and letters to the editor) not reporting original data were excluded. Evidence syntheses were excluded but their lists of included studies were screened for relevant constituent studies, to cross-check our own literature search.

Sources were screened in Covidence at the title/abstract and full-text level by two independent reviewers. Conflicts were resolved by a third reviewer or by consensus. Studies excluded at the full-text level are listed in the electronic supplementary material, Appendix S2, with reasons for exclusion.

### (c) Data extraction, risk of bias assessment and data analysis

Data extraction was completed in Microsoft Excel using a standardized data extraction form that is included in the electronic supplementary material, Appendix S3. Data were extracted by a single reviewer and verified independently by a second.

ROB was assessed using the ROB-2 tool for RCTs [9]. Observational studies were assessed using a modified version [10] of the ROBINS-I tool [11], which was appropriate for this rapid systematic review. The modified instrument is presented in the electronic supplementary material, Appendix S4. Each study was assessed by one reviewer and checked for accuracy by a second independent reviewer. Once a study met one criterion that made it ‘critical’ for ROB the assessment was stopped without completing it in full. ROB was not an exclusion criterion, but rather used as a tool for interpreting results of studies. The ROB tools are methods for identifying weaknesses in study design that fail to rule out bias, but they do not prove that bias exists.

We synthesized data narratively and in summary tables. We considered presenting the results as a meta-analysis, but ultimately deemed this inappropriate due to heterogeneity in study design, the variety of outcome measures, and poor reporting across the included studies. We did not carry out a formal GRADE assessment [12], but we considered the design of each study included in the review, the ROB, and the precision (confidence intervals) and direction of reported effects. Blobbograms were created to graphically display unpooled, unadjusted odds ratios (ORs) and 95% confidence intervals (CIs) for those studies reporting detailed data of SARS-CoV-2 infection events in those who wore a mask versus those who were unmasked, in those who wore a mask ‘sometimes’ versus those who were unmasked, or in those who wore a respirator versus a surgical mask. Blobbograms were created using Microsoft Excel.

## 3. Results

**Figure 1.**
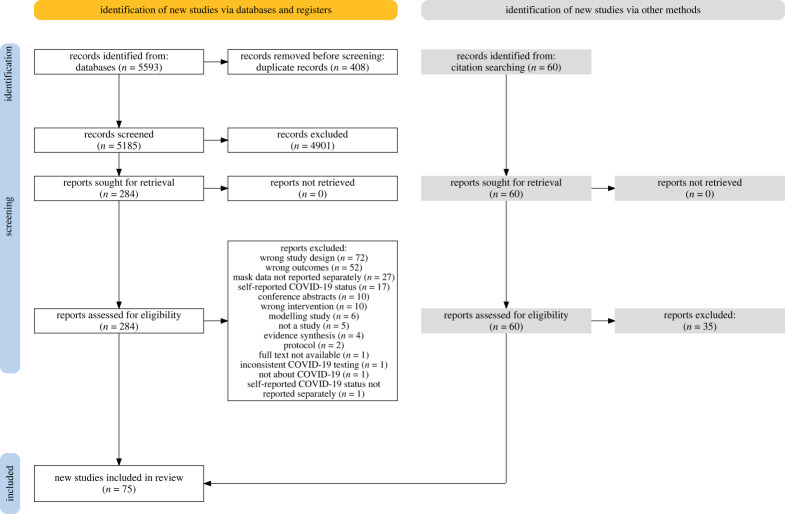
PRISMA 2020 Flow Diagram.

There were 5593 records identified by the database search, from which 408 duplicates were removed before screening. A total of 5185 records were screened at the title/abstract level, plus an additional 60 studies identified for screening from existing evidence syntheses. Of the 284 studies screened at the full-text level, 75 were included in this review. Figure 1 illustrates the screening process using a PRISMA 2020 Flow Diagram [13]. Table 1 summarizes the characteristics of included studies, with further detail including key findings presented in the electronic supplementary material, Appendix S5. Table 2 summarizes the number of studies addressing each type of comparison studied and the direction of their conclusions.

**Table 1.** High-level characteristics of included studies.

study	design	country	phenomenon of interest	outcome measure	mask-wearing measure
community settings					
Abaluck *et al*. [14]	RCT	Bangladesh	masks for reducing transmission; types of masks	serology (ELISA)	direct observation (weekly)
Andrejko *et al*. [15]	observational	USA	masks for reducing transmission; types of masks	molecular test (non-specific)	questionnaire
Andrejko *et al*. [16]	observational	USA	masks for reducing transmission	molecular test (non-specific)	interview
Areekal *et al*. [17]	observational	India	masks for reducing transmission	testing (method not specified)	contact tracing
Baig *et al*. [18]	observational	Pakistan	masks for reducing transmission	serology (CLIA or ELISA)	questionnaire
Boutzoukas *et al*. [19]	observational	USA	mask mandates for reducing transmission	testing (method not specified)	mandate data
Bundgaard *et al*. [20]	RCT	Denmark	masks for reducing transmission	PCR or serology (LFIA)	questionnaire
Cheng *et al*. [21]	observational	Hong Kong	masks for reducing transmission	PCR	interview
DeJonge *et al*. [22]	observational	USA	mask mandates for reducing transmission	testing (method not specified)	mandate data
Doung-Ngern *et al*. [23]	observational	Thailand	masks for reducing transmission; types of masks	RT-PCR	contact tracing
Doyle *et al*. [24]	observational	USA	mask mandates for reducing transmission	nucleic acid or antigen	mandate data
Gigot *et al*. [25]	observational	USA	masks for reducing transmission	saliva antibody testing	questionnaire
Goncalves *et al*. [26]	observational	Brazil	masks for reducing transmission	RT-PCR or antibody testing (non-specific)	interview
Hast *et al*. [27]	observational	USA	masks for reducing transmission	RT-PCR	contact tracing
Herstein *et al*. [28]	observational	USA	mask mandates for reducing transmission	testing (method not specified)	mandate data
Hobbs *et al*. [29]	observational	USA	masks for reducing transmission	RT-PCR	interview
Islam *et al*. [30]	observational	USA	mask mandates for reducing transmission	testing (method not specified)	mandate data
Jehn *et al*. [31]	observational	USA	mask mandates for reducing transmission	RT-PCR or NAAT or antigen	mandate data
Li *et al*. [32]	observational	USA	mask mandates for reducing transmission	testing (method not specified)	mandate data
Lio *et al*. [33]	observational	Macao	masks for reducing transmission	testing (method not specified)	questionnaire
Liu *et al*. [34]	observational	USA	masks for reducing transmission	RT-PCR (self-collected nasal)	questionnaire
Moek *et al*. [35]	observational	Germany	mask mandates for reducing transmission	RT-PCR	contact tracing
Nelson *et al*. [36]	observational	USA	masks for reducing transmission	testing (method not specified)	contact tracing
Pauser *et al*. [37]	observational	Germany	masks for reducing transmission	PCR	contact tracing
Payne *et al*. [38]	observational	USA	masks for reducing transmission	RT-PCR or seropositivity (ELISA)	questionnaire
Rebmann *et al*. [39]	observational	USA	masks for reducing transmission	RT-PCR (saliva)	contact tracing
Riley *et al*. [40]	observational	USA	masks for reducing transmission	testing (method not specified)	questionnaire
Shaweno *et al*. [41]	observational	Ethiopia	masks for reducing transmission	serology (chemiluminescent microparticle immunoassay (CMIA))	questionnaire
Sombetzki *et al*. [42]	observational	Germany	mask mandates for reducing transmission	PCR	mandate data
Sugimura *et al*. [43]	observational	Japan	masks for reducing transmission	PCR	interview
Theuring *et al*. [44]	observational	Germany	masks for reducing transmission	RT-PCR or serology (ELISA)	questionnaire
Ulyte *et al*. [45]	observational	Switzerland	mask mandates for reducing transmission	serology (multi-factorial seroprofiling (ABCORA))	questionnaire
van den Broek-Altenburg *et al*. [46]	observational	USA	masks for reducing transmission	PCR	questionnaire
Varela *et al*. [47]	RCT	Colombia	types of masks	RT-PCR or serology (rapid antibody test)	photograph confirmation of mask use; questionnaire; interview
Wang *et al*. [48]	observational	China	masks for reducing transmission	RT-PCR or serology (non-specific)	questionnaire
healthcare settings					
Adawee *et al*. [49]	observational	USA	mask mandates for reducing transmission	testing (method not specified)	mandate data
Aghili *et al*. [50]	observational	Iran	masks for reducing transmission; types of masks	PCR	questionnaire
Ambrosch *et al*. [51]	observational	Germany	mask mandates for reducing transmission	RT-PCR	mandate data
Belan *et al*. [52]	observational	France	types of masks	RT-PCR or antigen	questionnaire
Boffetta *et al*. [53]	observational	Italy	masks for reducing transmission	PCR	incident tracking system
Celebi *et al*. [54]	observational	Turkey	masks for reducing transmission	RT-PCR	questionnaire
Chatterjee *et al*. [55]	observational	India	masks for reducing transmission	qRT-PCR	interview
Chen *et al*. [56]	observational	China	masks for reducing transmission	qRT-PCR and serology (in-house enzyme immunoassay [EIA])	questionnaire
Collatuzzo *et al*. [57]	observational	Italy	masks for reducing transmission; types of masks	RT-PCR	incident tracking system
Coppeta *et al*. [58]	observational	Not reported	masks for reducing transmission	RT-PCR	interview
Davido *et al*. [59]	observational	France	masks for reducing transmission	RT-PCR	questionnaire
Doernberg *et al*. [60]	observational	USA	masks for reducing transmission	RT-PCR or serology (ELISA)	questionnaire
Gaikwad *et al*. [61]	observational	India	masks for reducing transmission	testing (method not specified)	incident tracking system
Gras-Valenti *et al*. [62]	observational	Spain	mask mandates for reducing transmission	testing (method not specified)	interview
Guo *et al*. [63]	observational	China	masks for reducing transmission	RT-PCR	questionnaire
Howard-Anderson *et al*. [64]	observational	USA	masks for reducing transmission	serology (ELISA)	questionnaire
Khalil *et al*. [65]	observational	Bangladesh	masks for reducing transmission	PCR	questionnaire
Khurana *et al*. [66]	observational	India	masks for reducing transmission	RT-PCR	interview
Kociolek *et al*. [67]	observational	USA	mask mandates for reducing transmission	PCR	mandate data
Kumar *et al*. [68]	observational	India	masks for reducing transmission; types of masks	RT_PCR	incident tracking system
Lai *et al*. [69]	observational	China	masks for reducing transmission	nucleic acid testing (method not specified)	questionnaire
Lentz *et al*. [70]	observational	Global	types of masks	PCR	questionnaire
Li *et al*. [71]	observational	USA	types of masks	PCR	interview
Loeb *et al*. [72]	RCT	Global	types of masks	RT-PCR	self-reported audits; diaries
Martischang *et al*. [73]	observational	Switzerland	types of masks	serology (CLIA or ELISA)	questionnaire
Mastan *et al*. [74]	observational	UK	types of masks	testing (method not specified)	questionnaire
Oksanen *et al*. [75]	observational	Finland	masks for reducing transmission	RT-PCR	questionnaire
Pan *et al*. [76]	observational	China	masks for reducing transmission	RT-PCR	questionnaire
Piapan *et al*. [77]	observational	Italy	masks for reducing transmission; mask mandates for reducing transmission	RT-PCR	interview
Rodriguez Lopez *et al*. [78]	observational	Colombia	types of masks	RT-PCR	questionnaire
Rolland *et al*. [79]	observational	France	mask mandates for reducing transmission	RT-PCR	mandate data
Sadeghi *et al*. [80]	observational	Iran	types of masks	RT-PCR	interview
Self *et al*. [81]	observational	USA	masks for reducing transmission	serology (ELISA)	questionnaire
Sertcelik *et al*. [82]^a^	observational	Turkey	masks for reducing transmission; types of masks	RT-PCR	interview or questionnaire
Shah *et al*. [83]	observational	USA	types of masks	RT-PCR	incident tracking system
Sims *et al*. [84]	observational	USA	masks for reducing transmission; types of masks	serology (ELISA)	questionnaire
Venugopal *et al*. [85]	observational	USA	masks for reducing transmission	PCR and serology (CMIA)	questionnaire
Wang *et al*. [86]	observational	USA	mask mandates for reducing transmission	RT-PCR	mandate data
Wang *et al*. [87]	observational	China	mask mandates for reducing transmission	molecular testing (non-specific)	mandate data
Wilson *et al*. [88]	observational	France	types of masks	PCR or antigen	questionnaire

^a^Non-peer-reviewed preprint.

**Table 2.** Effect of mask-wearing on SARS-CoV-2 transmission (number of studies of each type).

type of	type of	healthcare settings			community settings		
comparison	study	lower	no effect^a^	higher	lower	no effect^a^	higher
mask versus no mask	RCT	0	0	0	1	1	0
	observational	19	4	0	20	1	1
mask type (higher versus lower quality)	RCT	0	1	0	1	1	0
	observational	9	5	0	1	1	0
mask mandate	RCT	—	—	—	—	—	—
	observational	7	1	0	9	1	0
sum		35	11	0	32	5	1

^a^No statistically significant effect.

There were 35 studies set in the community (three RCTs (14–16), 32 observational studies [15–19,21–46,48] and 40 studies situated in healthcare settings including hospitals and long-term care facilities (LTCFs) (one RCT [72], 39 observational studies [49–71,73–88]).

The United States was the most frequent host country, presenting 18 community-based studies and 10 healthcare-based studies, totalling 37% of all included studies. In community-based studies, Germany was the next most frequent host country (*n* = 4 studies), with the remainder coming from a variety of other countries. In healthcare-based studies, China (*n* = 5), France (*n* = 4) and India (*n* = 4) were common settings, followed by Italy (*n* = 3), Turkey (*n* = 2), Iran (*n* = 2) and two multinational studies. One study did not report its host country.

Studies with data collection taking place early in the pandemic (defined in this review as December 2019–July 2020) were far more common in healthcare settings (*n* = 30/40; 75%) than in community settings (*n* = 9/35, 26%). Most community-based studies took place from mid-2020 to late 2021 (*n* = 25/35; 71%). Only four studies (5%), three in the community and one in healthcare, reported on data collected during the Omicron era of the pandemic (November 2021–January 2023).

PCR was the most common method of testing for SARS-CoV-2 infection (*n* = 47/75; 63%), followed by serology (*n* = 18/75; 24%). Thirteen studies (17%) were not specific about the testing method used, only stating that COVID-19 cases were laboratory-confirmed or drawn from a database of confirmed infections. Enzyme-linked immunosorbent assay (ELISA) was the most common immunoassay used (*n* = 9/18; 50% of studies using serology; table 1).

Most observational studies of mask-wearing relied on self-reported mask-wearing data from participants. Thirty-three studies (44%) used questionnaires to collect this information, while 13 (17%) used interviews and seven (9%) used contact tracing methods that were not further described. Studies of mask mandates typically used publicly available data about whether mask mandates were in effect (*n* = 14/18; 78%). Five studies in healthcare settings were able to draw information about mask-wearing from COVID-19 incident tracking systems.

Of the 22 community-based observational studies of masks in general and types of masks, 14 studies (64%) asked whether participants wore masks but did not ask whether masks were worn by potential sources of infection. Four studies asked if either party had been masked, three asked if both parties had been masked and one focused solely on the mask-wearing of the COVID-19-positive contact. In healthcare settings, mask-wearing by healthcare workers (HCWs) and not by patients was reported in all but one study, which focused instead on mask-wearing by LTCF residents [50]. Six studies reported on mask use by both HCWs and patients, one of which [57] focused specifically on COVID-19 patients. The majority of studies evaluated whether individual mask wearers were protected from SARS-CoV-2 infection, but studies that measured effects in whole populations (e.g. cluster randomized trials, communities under mask mandates) did not distinguish whether transmission was reduced from infected mask wearers, to uninfected mask wearers, or both (electronic supplementary material, Appendix S5).

ROB was consistently high across all included study designs. All included RCTs were at ‘high’ ROB. All observational studies in healthcare settings (*n* = 39) and most studies in community settings (*n* = 29/32; 91%) were at critical ROB in at least one domain. Of the remaining three observational studies, two were at serious ROB [30,32] and one was at moderate ROB [15]. Critical ROB in observational studies was often related to study authors' inability to definitively relate outcomes to masks or mask mandates alone (*n* = 30/68; 44% of critical assessments) or due to a failure to adjust for other COVID-19 protective interventions either before or during the study period (*n* = 11/68; 16%).

### (a) Masks for reducing transmission of SARS-CoV-2

Forty-seven studies reported on the effectiveness of masks for reducing transmission of SARS-CoV-2 as their primary outcome: 24 studies (two RCTs, 22 observational) in community settings [14–18,20,21,23,25–27,29,33,34,36–41,43,44,46,48] and 23 observational studies in healthcare settings [50,53–61,63–66,68,69,75–77,81,82,84,85].

Of the two RCTs conducted in community settings, one cluster RCT [14] found a 9.5% reduction in symptomatic seroprevalence (*n* = 105 fewer symptomatic seropositives; adjusted prevalence ratio [aPR] 0.91 [95% CI: 0.82, 1.00]) and an estimated 11.6% reduction in the proportion of individuals with COVID-19-like symptoms (*n* = 1541 fewer people reporting symptoms; aPR 0.88 [95% CI: 0.83, 0.93]) in communities where masks were distributed and their use promoted compared with control communities. In the other RCT [20], infection with SARS-CoV-2 occurred in 1.8% of participants in the mask group (recommended to wear medical masks) versus 2.1% in the control group (OR 0.82 [95% CI: 0.54, 1.23]), with the authors concluding that the difference was not significant. Of note, the latter RCT took place early in the pandemic (April–June 2020) and only 95 out of 4862 (2%) of participants who completed the study were infected with SARS-CoV-2, and self-reported adherence to mask-wearing among participants was poor.

Of the 45 observational studies, 39 (87%) found that mask-wearing was associated with a reduction in SARS-CoV-2 transmission. These 45 studies had a wide variation in study design and intervention characteristics, and almost all (*n* = 42; 91%) relied on self-reported mask-wearing data from participants. Five studies concluded that masks had no significant effect on transmission, and one favoured the control group. Notably, the study favouring controls [18] is one of the three preprints included in this review; it had not been peer reviewed nor subsequently published, and the data presented are inconsistent with the authors' own conclusion that masks were protective.

**Figure 2.**
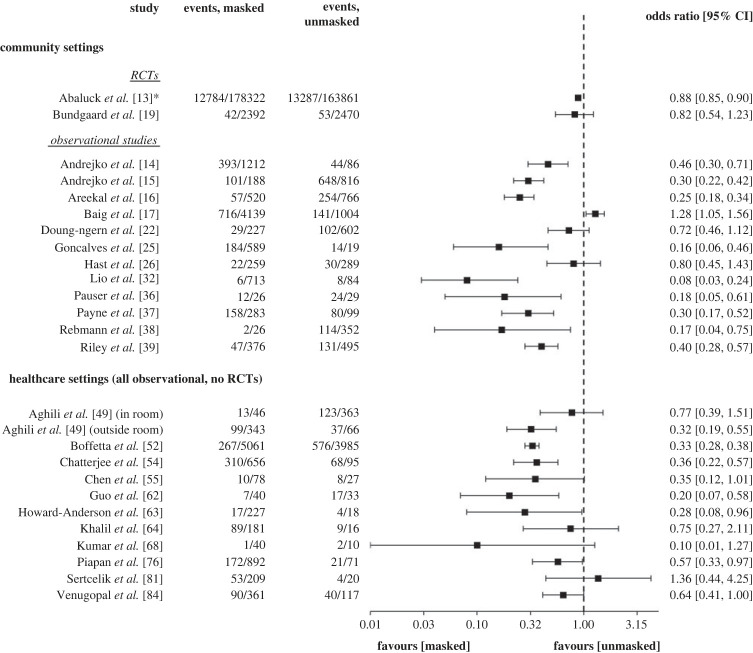
ORs and 95% confidence intervals of a subset of eligible included studies comparing masked versus unmasked. *Although Abaluck *et al*. [14] is a cluster RCT, the sample sizes presented in this figure represent events at the individual level.

A subset of 24/47 studies (two RCTs, 22 observational; 14 in community settings, 10 in healthcare settings) reported the number of SARS-CoV-2 infection events in those who wore a mask versus those who were unmasked. The unpooled, unadjusted ORs and 95% CIs of these studies are graphically displayed in figure 2. The blobbogram illustrates that there were only two RCTs, and their effect sizes were smaller and within a closer range than most observational studies. The figure helps to illustrate between-study comparisons in direction of effect while also making explicit the existing sampling imbalance and wide CIs across many of the studies.

**Figure 3.**
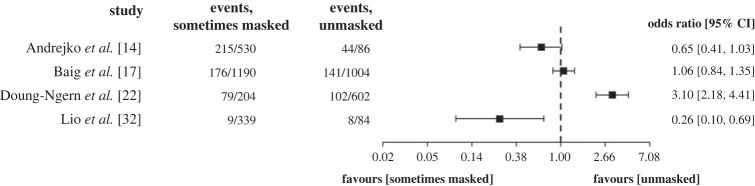
ORs and 95% confidence intervals of a subset of eligible included studies comparing sometimes masked versus unmasked.

A further subset of four studies, all observational and set in the community, reported the number of SARS-CoV-2 infection events in those who self-reported wearing a mask ‘sometimes’ versus those who were unmasked. The unpooled, unadjusted ORs and 95% CIs of these studies are visualized in figure 3. Two studies showed a positive association and two showed a negative association of sometimes wearing a mask with risk of SARS-CoV-2 infection.

In summary, the great majority of studies found that masks (*n* = 39/47; 83%) reduced transmission, although the magnitude of measured effects was variable and the quality (precision and ROB) of evidence in both community and healthcare settings was low.

### (b) Comparative effectiveness of different types of masks

Nineteen studies compared the effectiveness of different types of masks for reducing transmission of SARS-CoV-2: four studies (two RCTs, two observational) in community settings [14,16,23,47] and 15 studies (one RCT, 14 observational) in healthcare settings [50,52,57,68,70–74,78,80,82–84,88].

Two RCTs compared different types of masks in community settings. In the implementation cluster RCT cited above [14], surgical masks were associated with an 11.1% reduction in symptomatic seroprevalence (aPR 0.89 [95% CI: 0.78, 1.00]) compared with no statistically significant effect of cloth masks (aPR 0.94 [95% CI: 0.78, 1.10]). The other RCT [47] compared use of a closed face shield with surgical face mask to using a surgical mask alone to prevent SARS-CoV-2 infection. Only one participant tested positive for SARS-CoV-2 in the mask-only group versus three participants in the mask plus shield group. In the per-protocol analysis, the absolute risk difference was –1.4% (95% CI: –4.2%, 1.4%), indicating non-inferiority of the mask plus shield.

Of the observational studies in community settings, one study [16] found that N95 or KN95 masks and surgical masks were effective while cloth masks were not, but the other [23] found that type of mask was not significantly associated with infection risk. Both studies relied on self-reported mask-wearing data from participants without any attempts to observe adherence.

In healthcare settings, one non-inferiority RCT [72] compared different types of masks (fitted N95 respirator versus surgical mask) used by HCWs in hospital settings in Canada, Egypt, Israel and Pakistan. The authors identified an infection rate across all sites of 10.5% in the medical mask group compared with 9.3% in the N95 group (hazard ratio 1.14 [95% CI: 0.77, 1.69]), indicating that surgical masks were non-inferior to N95 respirators. Among participants who contracted RT-PCR-confirmed COVID-19, two participants were hospitalized in the medical mask group compared with one in the N95 group. Notably, data collection in Egypt took place when the highly transmissible Omicron variant was in heavy circulation. This contrasts with data collection in Canada that took place early in the pandemic. Furthermore, mask-wearing outside of work was not measured.

Of the 14 observational studies comparing different types of masks in healthcare settings, seven found that respirators were associated with a decreased risk of transmission compared with other masks and five found that there was no statistically significant difference between the two types. One study [88] compared respirator versus surgical mask use during AGPs for COVID-19 versus non-COVID-19 patients and found that while respirators were associated with lower infection rates than surgical masks in HCWs performing AGPs and non-AGPs for COVID-19 patients, the opposite was true for those caring for non-COVID-19 patients, for whom wearing a respirator was associated with a higher risk of infection than a surgical mask.

**Figure 4.**
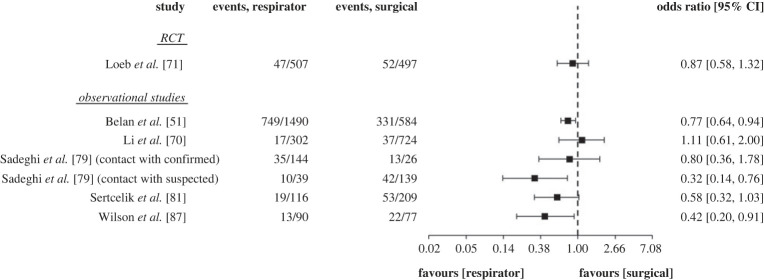
ORs and 95% confidence intervals of a subset of eligible included studies comparing respirator versus surgical masks.

A subset of six studies in healthcare settings (one RCT, five observational) reported the number of SARS-CoV-2 infection events in those who wore respirators versus surgical masks. The unpooled, unadjusted ORs and 95% CIs of these studies are visualized in figure 4. The figure illustrates the variation in this evidence, with several 95% CIs crossing the line of no effect.

In summary, where significant effects were reported, they favoured wearing higher quality rather than lower quality masks. However, the majority of studies suffered from a critical ROB in at least one domain, and effects were uncertain in magnitude and variable betweenstudies.

### (c) Mask mandates for reducing transmission of SARS-CoV-2

Eighteen observational studies reported on the effectiveness of mask mandates for reducing transmission of SARS-CoV-2: 10 in community settings [19,22,24,28,30–32,35,42,45] and eight in healthcare settings [49,51,62,67,77,79,86,87]. Sixteen of these studies (89%) found that mask mandates were associated with a reduction in transmission, while two found that they had no significant effect on transmission.

Six of the 10 community-based studies were set in schools. The majority of studies (*n* = 9/10; 90%) in the community found mask mandates to be associated with a reduced risk of SARS-CoV-2 transmission. The only community-based study to find mask mandates had no significant effect on transmission [35] studied transmission on airplanes before and after the implementation of a universal mask mandate. This retrospective study was carried out before Omicron and included 95 participants from 46 flights, among which there were only four instances of probable in-flight transmission (two before the mandate, and two after). Adherence to the mandate was not measured in this study, nor was it measured in any other study of mask mandates.

All hospital-based studies of mask mandates (*n* = 7) found mask mandates to be associated with lower incidence of SARS-CoV-2 infection. By contrast, findings from the only study based in LTCF indicated no significant effect of mask mandates on transmission [79].

In summary, the majority of included studies found that mask mandates reduced transmission of SARS-CoV-2, albeit with effects of variable magnitude and low precision. The quality of the evidence supporting mask mandates was low based on ROB assessments and heterogeneity in study designs.

## 4. Discussion

Most of the studies included in this review favoured the wearing of masks, of wearing higher quality masks (respirators) and mask mandates to reduce SARS-CoV-2 transmission (table 2). Sample size, intervention implementation and measurement, measures of infection, and effect size all varied greatly across studies, and 95% (*n* = 71/75) were performed before the emergence of highly transmissible Omicron variants, which began in November 2021. RCTs addressing the three review questions were rare and were all assessed to be at high ROB. Although few in number, the two RCTs assessing the effectiveness of mask use produced lower effect sizes (unadjusted OR 0.82 to 0.88, consistent with a 12–18% reduction) than observational studies, which had a wide range (unadjusted OR 0.08 to 1.28, consistent with a 92% decrease to 28% increase). Observational studies made up 95% of included studies and were almost all at critical ROB in at least one domain. Laboratory-confirmed SARS-CoV-2 was a required outcome measure for inclusion in this review, and while most studies were specific about the type of test used, 17% did not report this information. How mask-wearing was recorded varied among studies and typically relied on self-reporting by study participants. The results of all included studies apply under the conditions in which the studies were carried out and caution is needed when generalizing from them. For instance, findings related to the effectiveness of mask mandates do not mean that mask mandates are always effective or appropriate.

Studies carried out in healthcare settings were among the first to contribute to the body of literature examining whether masks reduce the transmission of SARS-CoV-2 infection, with 75% of included healthcare studies occurring prior to July 2020. This is likely due to the implementation of masks in these settings at the very beginning of the pandemic before recommendations and mandates were extended to the public. While the timing makes these early studies less subject to confounding due to vaccines and other pharmaceutical interventions, they pre-date the emergence of several SARS-CoV-2 variants of concern, so it is not clear whether masks are effective, for example against highly transmissible Omicron variants. Additionally, masks in these settings were usually introduced in conjunction with other NPIs including personal protective equipment, visitor restrictions, strict quarantine and isolation rules, and in some cases, increased ventilation requirements in COVID-19 wards. Likewise, masks were rarely the only NPIs implemented in community settings, particularly early in the pandemic when people were encouraged to limit mobility, self-isolate when ill or in contact with cases and seek frequent testing. Regarding bias, there is a risk of overstating the effectiveness of masks if, for example, mask wearers were more cautious about meeting others in their communities. On the other hand, the potential effectiveness of masks could be understated if the risk of infection has been reduced by other concurrent NPIs, as suspected by Bundgaard *et al*. [20].

Because it is difficult to monitor mask-wearing in practice during a health emergency, the observational studies included in this review have relied on self-reported mask-wearing as a measure of mask use. Some studies made efforts to guard against recall bias by following participants prospectively and following up on positive COVID-19 results as soon as possible, thereby reducing the chance of such bias. This approach was more common in healthcare-based studies than those set in the community. Standardized methods for recording and reporting adherence to masking are needed, as is increased rigour in reporting the exact designs and outcomes of observational research.

There are a number of considerations and challenges inherent in designing high quality trials at the individual or population level to study the effectiveness of health interventions such as masking or adherence to mask mandates. This is particularly true when different actors with different experiences and varying environmental contexts behave in unpredictable ways that are difficult to measure consistently. Understanding if face masks are effective in reducing the transmission of SARS-CoV-2 is contingent on the intervention (masks or mask mandates) being delivered in a similar way and on study participants wearing masks in a similar way. Repeated-occurrence behaviours such as masking are known to vary from day to day at the individual level in response to environment/contextual and intra- and inter-individual factors [89]. These factors can influence recruitment, retention and outcome measures. Attention to and evaluation of trial process interventions and anticipation of potential barriers to adherence could enhance future trials [90].

Our review expands upon existing reviews by including more recent studies (released up to 27 January 2023), by broadening inclusion criteria to encompass a range of study designs and interventions, and by including evidence from community and healthcare settings. It also differs from Jefferson *et al*.'s and Chu *et al*.'s reviews in being restricted to COVID-19-related studies. The rates of severe infection and mortality from COVID-19 infection were significantly higher than those of influenza across age ranges, and initial estimates of the epidemiologic reproductive number established that SARS-CoV-2 is more transmissible than influenza. We therefore decided against directly comparing COVID-19 studies with studies of other illnesses and excluded non-COVID-19 studies from this review.

As this was a rapid systematic review, we abbreviated established systematic review methodology. We did not attempt to classify studies according to whether the masks reduced the transmission of infection from infected mask wearers to others, to uninfected mask wearers from other infected people, or both. We stopped our ROB assessments once a study received a critical judgement in at least one domain. Thus, we did not fully grade the certainty or validity of the evidence, and this limits our ability to estimate the impact of these studies' ROB on the certainty of their findings. However, it is important to note that a critical assessment in one domain did not necessarily mean that bias was detected, but rather that it could not be ruled out. Additionally, although not typically recommended for systematic reviews, the search for this rapid review included keywords related to outcomes of interest (e.g. ‘transmission’; ‘spread’). While this kept the number of results at a feasible level for rapid screening, relevant articles not reporting on these concepts in the title, abstract or subject headings were not captured. We compensated for this limitation by thoroughly screening the included studies of other systematic reviews related to our topic.

## 5. Conclusion

Most of the studies included in this rapid systematic review were observational rather than experimental. Study designs commonly suffered from a critical ROB. The effects measured in each study were variable in magnitude and generally of low precision. Nevertheless, taking together the evidence from all studies, we conclude that wearing masks, wearing higher quality masks (respirators), and mask mandates generally reduced the transmission of SARS-CoV-2 infection.

### (a) Patient-identified key messages

Patient Partners on this project were invited to identify important findings from this synthesis work. Patients and families, particularly those with compromised health, worried about how the limited level of evidence supporting the use of masks to reduce transmission of SARS-CoV-2 will impact adherence, particularly in community settings.

## Data Availability

Supporting information and the data are provided in the electronic supplementary material [91].
